# A66 THE IMPACT OF A MULTIDISCIPLINARY ADOLESCENT AND YOUNG ADULT (AYA) INFLAMMATORY BOWEL DISEASE (IBD) ON CLINIC NO SHOW RATES

**DOI:** 10.1093/jcag/gwab049.065

**Published:** 2022-02-21

**Authors:** R Sharma, S L Halder, M Zachos, C Radoja, C Grant, U Chauhan, E Brackenridge, R Issenman, M Sherlock, J K Dowhaniuk, N Pai, H Brill, E Ratcliffe, N Narula, J Marshall, K Prowse

**Affiliations:** 1 McMaster University, Hamilton, ON, Canada; 2 Hamilton Health Sciences, Hamilton, ON, Canada

## Abstract

**Background:**

Transitioning from pediatric to adult health care is associated with significant psychosocial and clinical morbidity. Adolescents not only transition their medical care, but also experience vast changes in the physical, social, and psychological spheres of their lives. The medical team must help navigate these changes to provide optimal care.

IBD in adolescence is associated with increased hospitalizations and surgery. This is due to several factors, including medication non-adherence and a failure to attend medical appointments. There has been a greater focus on improving care for this unique population.

McMaster Children’s Hospital has integrated the AYA IBD clinic for patients between the ages of 16 and 22. The goal is to transition patients using a developmentally appropriate framework to facilitate self-efficacy and help identify comorbid mental health conditions while building resilience.

**Aims:**

To explore the impact of the implementation of a dedicated transition clinic on attendance at medical visits for AYA patients with IBD.

**Methods:**

The total numbers of patients booked in the AYA IBD Clinic was compared to an age matched subset of the patients in the adult McMaster Complex IBD (CIBD) Clinic. These visits were assessed based on whether the visit was: attended, cancelled, or no showed. Visits were then stratified between in-person and virtual visits. Unpaired *t* tests was performed to compare the AYA IBD clinic and the CIBD clinic. Findings were deemed significant based on p-values <0.05.

**Results:**

The percentages of patients that attended visits (in-person or virtually) was similar between both clinics at 86% versus 79% Year 1 (Y1) and 76% versus 81% Year 2 (Y2). The number of patients seen in the AYA clinic increased from Y1 (n=92) to Y2 (n=131). The CIBD clinic saw fewer patients between Y1 (n=202) and Y2 (n=79). There were a higher number of patients who cancelled or no showed in Y2 versus Y1 for the AYA virtual visits (13 versus 8) compared to the CIBD clinic (Y2,1 versus Y1,1).

**Conclusions:**

Our results highlight the challenges of transitioning adolescent patients with IBD. Our retrospective study was not powered to show significance. Given the increase in cancellation and no-show rates in Y2, the AYA clinic has incorporated a patient navigator to issue reminder phone calls and facilitate communication with patients between clinics. Future studies will re-assess how the presence of a patient navigator impacts attendance and cancellation rates. Future studies will also assess how the AYA clinic impacts transition readiness and self-efficacy, which is being measured through validated questionnaires in our clinic.

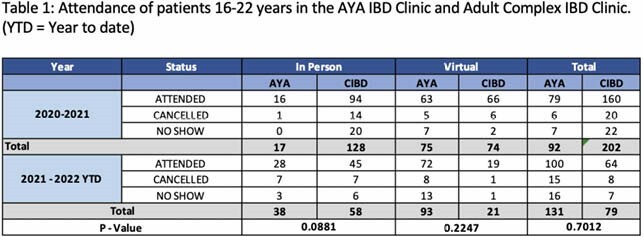

**Funding Agencies:**

Grants-In-Aid

